# Initial Development of the Activity Card Sort-Advancing Inclusive Participation from a Homeless Population Perspective

**DOI:** 10.1155/2020/9083082

**Published:** 2020-07-03

**Authors:** Quinn P. Tyminski, Ronald R. Drummond, Claire F. Heisey, Shelby K. Evans, Audra Hendrix, Lisa A. Jaegers, Carolyn M. Baum

**Affiliations:** ^1^Program in Occupational Therapy, Washington University School of Medicine, St. Louis, Missouri, USA; ^2^Department of Occupational Science and Occupational Therapy, Saint Louis University, St. Louis, Missouri, USA

## Abstract

**Methods:**

Develop a new version of the Activity Card Sort-Advancing Inclusive Participation to include occupations experienced by the homeless population, including nonsanctioned occupations. This study occurred in two phases: (1) tool development (item selection, content expert review, line development drawing, and assessment of content validity) and (2) tool use to determine face validity. Participants were selected through a convenience sample at a local homeless shelter and academic institution. Participants experiencing homelessness (phase 1: *N* = 13, phase 2: *N* = 10) were required to be seeking services at the homeless shelter, while nonhomeless participants (phase 2: *N* = 30) worked full-time, resided with a significant other, and had personal transportation.

**Results:**

An assessment of 76 occupations, corresponding line drawings, and follow-up questions was created. An initial construct validity study demonstrated differences between occupational participation of those who are homeless and nonhomeless in the areas of social engagement, nonsanctioned occupations, work and education, and home management. Both groups reported previous, current, or desired engagement in the occupations identified in the assessment. *Conclusions and relevance.* The purpose of this study was to create an inclusive assessment for use in the homeless population and complete a construct validity study of the assessment tool. Although the results indicated some differences in the frequency with which occupations were performed, the results demonstrated that all individuals participate in occupations that many not contribute to their health and wellness. This initial work supports the future development of a tool that is inclusive of all occupations to obtain a holistic picture of an individual's participation.

## 1. Background

Nonsanctioned occupations, or those that are not socially accepted as normative, still hold value, meaning, and purpose to those performing them and can provide a sense of camaraderie, means of escape from difficult circumstances, or an emotional outlet [1]. Omitting these occupations during initial assessment may lead to an incomplete understanding of people's roles, routines, and habits of engagement, resulting in interventions that may not address the needs of the client [2]. It is essential to address nonsanctioned occupations to ensure a holistic and accurate representation of the client, as an understanding of participation in these occupations serves to inform and guide treatment and ensure client-centered care [1]. There is a need for an evaluation tool that allows for greater respect to diversity, inclusion, and participation in all desired occupations in order to obtain a holistic representation of occupational participation of people who engage in occupations that may threaten their families, work, or community participation.

In 2017 in the United States, there were approximately 554,000 individuals experiencing homelessness (IEH) [3]. IEH experience occupational disruption due to a variety of environmental and social barriers that limit their choice and autonomy related to meaningful participation [1, 4]. External barriers including policy, social stigma, low socioeconomic status, and rigid shelter rules often limit participation in meaningful activities for IEH. A recent study reported that the lack of participation in occupations and resultant boredom affected IEH in such an extreme way that they prioritized engagement in meaningful occupations over more tangible needs such as housing [4].

There are many barriers to and minimal opportunities for meaningful participation in homeless shelters and community-based homeless services. Homelessness has become an emerging practice for occupational therapists to improve health and well-being through occupational performance and participation interventions [5–7]. Occupational therapy practitioners play a major role in evaluating and facilitating independence and well-being in IEH. They also contribute an occupation-based perspective to the multidisciplinary treatment teams in emergency shelters and supportive housing programs [7–9].

Occupational therapists need a tool to address issues of client choice, autonomy, dignity, and identity in IEH [10]. While this role for OT is still emerging in the homeless population, there is a need for a tool to assist practitioners and clients to engage in a discussion around sensitive occupations and lead to client-centered plans of care.

The Activity Card Sort (ACS) was designed for use with community-dwelling older adults to inform the development of therapy goals by identifying changes in occupational participation as a measure of health and well-being and to create an occupational history to understand the impact of loss of health, home, and resulting trauma on their lives [11]. The ACS has shown high reliability and validity with older adults, community-dwelling adults, and persons with cancer and Parkinson's disease [12–16]. It has been adapted in more than a dozen unique populations of varying ages, cultures, and languages. The Activity Card Sort-Advancing Inclusive Participation (ACS-AIP) is a derivative of the original ACS and includes some of the core occupations in which all individuals engage or the homeless population may have done before. The authors had permission from the originator of the ACS to develop this version.

To inform best-practice intervention, an accurate occupational profile of the client, including nonsanctioned occupations, is necessary to provide a comprehensive picture of occupational participation [5]. Like other versions of the ACS, the assessment may be improved with greater sensitivity to gender, ethnicity, socioeconomic status, and age diversity of the intended population in item selection and pictorial representation [12].

The need for a valid assessment to inform intervention for IEH lends itself to the development of this version of the ACS-AIP, targeted to understand the unique occupational participation of the homeless population. A standardized assessment will serve as a tool to demonstrate occupational participation in a population of IEH; such a tool will facilitate interviews and goal creation and lead to targeted client services to increase participation in meaningful occupations.

## 2. Methods

The creation of the ACS-AIP occurred in two phases: (1) tool development through iterative steps of testing including item selection, content expert review, photograph and line development drawing, and photo captioning and assessment of content validity; and (2) tool use to determine feasibility and construct validity. The university's internal review board approved all stages of participant engagement.

### 2.1. Phase 1: Tool Development

Preliminary item selection was completed through a literature review of occupations in which IEH participate. The following search terms were used: occupation-based assessments, nonsanctioned occupations, occupational participation of IEH, and occupational justice (Table 1). In addition to the literature, occupations from the original version of the ACS, the Vineland Adaptive Behavior Scales, and a shelter-based assessment entitled the Vulnerability Index-Service Prioritization Decision Assistance Tool (VI-SPDAT) were included. An interview regarding time use and occupational participation with an IEH at a local shelter was also conducted, and the identified occupations were included in the preliminary list. Preliminary item selection resulted in 347 occupations. Duplicate or similar occupations were condensed, leaving 197 unique items for expert review.

Next, the list of 197 occupations was provided to six content experts. Two occupational therapy assistants and four occupational therapists working at organizations serving IEH completed a Delphi survey to provide both qualitative and quantitative feedback [23]. Experts ranked each occupation on an ordinal scale based on how often they estimated their clients participated in each occupation (i.e., daily, often, sometimes, rarely, never). Experts provided qualitative feedback through follow-up questions regarding any missing occupations, the names of the occupations, and any occupations with potential for retraumatization. Following the survey, each item was ranked based on the frequency of participation reported by content experts. Qualitative data were utilized to determine whether any items needed to be added, changed, or removed.

#### 2.1.1. Results Phase 1

**Figure 1:**
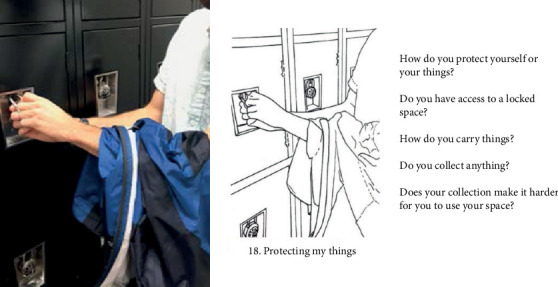
Activity Card Sort-Advancing Inclusive Participation card number 18: Protecting my things.

Based on feedback from expert reviewers, six occupations were added to the original list of 197 items, 17 were reworded, 13 were removed due to redundancy, and 114 highly specific items were removed. Examples of occupations ranked as daily participation were using public transport, smoking cigarettes, and managing a family. Examples of suggested occupations to add include managing pregnancy, menstrual hygiene management, and engaging with the legal system. Finally, reviewers suggested the removal of some items due to retraumatization such as self-harming behaviors. The resulting list included 85 occupations. Next, photographs were taken and converted by hand into line drawings, which were then made into the assessment cards (Figure 1). Written consent was obtained for producing photographic images.

Content validity was obtained using a convenience sample (*N* = 13) of adult participants receiving services at a local emergency homeless shelter. After demographic information was obtained (Table 2), participants were presented each card without the written caption and were asked to name the activity depicted. After each response, the participant was verbally given the caption and asked whether they thought it depicted the activity.

Following participant feedback, 28 captions were reworded, ten items were removed, six new photographs and drawings were taken to better depict the activity, 32 drawings were edited to increase clarity, and one activity was added. The resulting assessment included 76 validated line drawings, as well as, several follow-up questions for each occupation to be used by the clinician to collect further information (see Figure 1).

Follow-up questions were finalized for each of the 76 occupations to guide therapists in deepening the conversation. Categories for sorting were established based on the language in the Occupational Therapy Practice Framework to allow therapists to discover patterns within domains of occupation [5]. These categories include activities of daily living, home management, nonsanctioned occupations, physical activity, work and education, leisure, social participation, and health management [5]. Activities of daily living were added to this version of the ACS, as they were important to this population and were not included in other versions of the assessment.

#### 2.1.2. Discussion Phase 1

The ACS-AIP was developed to explore the unique occupational participation of the homeless population. First published by Twinley [2] under the term “the dark side of occupation” and later coined “non-sanctioned occupations” by Kiepek, Beagan, Laliberte Rudman, and Phelan [24], several articles have discussed the urgency for occupational therapy practitioners to address such occupations to reduce stigma and avoid bias. Creating an ACS that includes nonsanctioned occupations can bring focus to those occupations that are frequently not acknowledged. By beginning the discussion of less-recognized occupations in the evaluation process, occupational therapy practitioners can encourage rapport surrounding difficult topics, reduce stigma regarding certain occupations, and ensure that they are providing treatments that are holistic, client-centered, and meet the needs of the client.

This version differs from previous iterations of the ACS as it uses line drawings to reduce or remove indications of specific race, ethnicity, gender, and sex [25]. The choice to make this change was based on recent literature regarding the changing demands for occupational therapy practitioners working in community-based settings [26]. The use of line drawings to depict occupations is a new contribution in this version of ACS. Photographs were converted to line drawings to remove any notions of gender, race, ethnicity, age, and environment and were purposefully adjusted to exclude seasonal or geographic representations. This decision to remove potentially identifying features was prompted by AOTA's Vision 2025, which states that occupational therapy services should be accessible to all, with a specific focus on culturally responsive and highly customized service [27]. Therefore, the ACS-AIP pilot removed cultural indicators for this initial testing and will consider tailoring it to particular cultural and/or ethnic groups in the future.

Another contribution of this iteration is the inclusion of follow-up questions for each card, located on the back of each drawing (see Figure 1). The inclusion of follow-up questions provides a tool for clinicians to further prompt conversations and treatment planning. These questions help the assessor to explore the details of the occupations with the client and to frame treatment planning. It is not expected that each follow-up question will always be asked for each occupation; rather, the questions may serve to guide the discussion and to determine whether or when more information is needed.

The ACS-AIP is unique in three distinct ways: the inclusion of nonsanctioned occupations, use of inclusive line drawings, and addition of guided discussion questions. Thus, the title of this version was chosen to reflect the ability of this assessment to make progress in addressing inclusivity in the skills, activities, and communities from which our clients derive meaning. The term “advancing” recognizes the dynamic nature of occupation as it interacts with culture and society. Inclusion has become an important concern among occupational therapy practitioners and serves to ensure adequate representation. Participation as described by Law [28] is the act of sharing or taking part in something. The ACS-AIP adds this dimension to the management of community-based populations by actively acknowledging the biases surrounding nonsanctioned occupations that are part of engagement for IEH. For this population, participation is not only the desired outcome; it is also the process by which the outcome is achieved.

#### 2.1.3. Limitations Phase 1

The initial development of this assessment had several limitations that should be recognized. Some occupations such as exercising, gardening, and going to family gatherings were included despite low rankings of participation during the content expert review. These were selected because they are activities people may have previously done and may give insight into future interest and treatment activities. The development of items was focused on a specific subpopulation of mostly adult males residing in a local homeless shelter; items were not trialed in the general population. A small sample of content experts and participants in the community were engaged to develop initial content validity and to refine items. The sole use of healthcare providers to serve as content experts many have caused an unintentional bias in some of the occupations included in this assessment. Finally, the authors plan to engage with initial users and will use their input in the design of a study to include more participants, a wider geographic representation, and add additional occupations, if necessary.

### 2.2. Phase 2: Tool Use: Face Validity

To determine, the feasibility and construct validity of the ACS-AIP was completed as the initial step in developing this tool. A convenience sample of 30 participants was recruited for three subgroups: (1) IEH, (2) staff at the homeless shelter, and (3) faculty and staff at a local academic institution. Each subgroup consisted of 10 participants. Written consent was obtained prior to each interview. Inclusion criteria for IEH participants included clients seeking services at the homeless shelter. Inclusion criteria for nonhomeless participants included: full-time (40 hours per week) employment, private transportation, and residence with a significant other as seek perspectives from those with different lifestyles than IEH participant (*N* = 30) demographic information can be found in Table 3.

**Figure 2:**
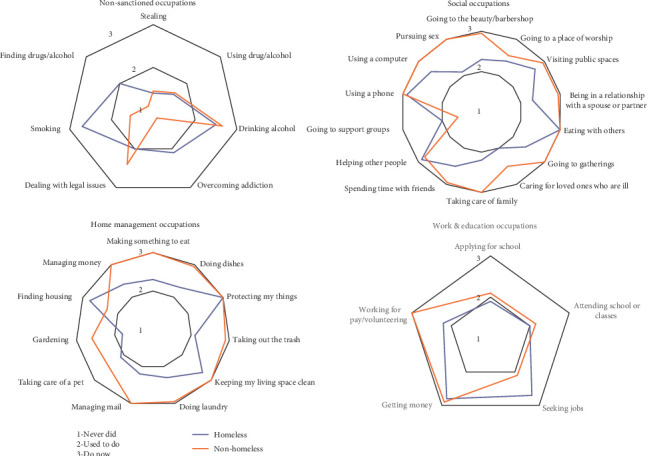
Radar graphs of occupational participation among homeless and nonhomeless groups.

Following the completion of the demographic information, each participant completed the ACS-AIP by sorting the 76 cards into categories of “never did” (1), “given up” (2), or “do now” (3) (see Figure 2) in order to determine whether the occupations in the ACS-AIP represent the daily life experiences of these groups. Qualitative data were also collected through a semistructured interview following the administration of the ACS-AIP. The interview asked participants three follow-up questions (“Were there any things that you do on a daily basis that were not present in these cards?” “Did you feel uncomfortable or upset by being asked about any of the activities in the cards?” “What is your opinion of this activity?”). All nonhomeless participants were asked two additional follow-up questions regarding the usefulness of the assessment (“What types of settings do you feel this assessment tool might be useful in?” “What are your overall opinions of this assessment?”). Each session lasted for approximately 1 hour, and each administration of the assessment was videotaped for accuracy and later destroyed.

Quantitative data obtained were analyzed using the Statistical Package of Social Sciences 24 [29]. For each area of occupation, the average answer was calculated and then rounded to the closest whole number to associate with each answer. Based on the results of data, participants from the academic institution and those from the shelter staff were recategorized into a single “nonhomeless participants” group, as there were similar patterns of participation between the two groups. Figure 2 depicts the average participation of the two participants groups (i.e., nonhomeless participants and IEH). Using spider graphs, Figure 2 demonstrates the differences in participation between IEH and nonhomeless participants. By coding the “never did” sorting category as “1,” located in the middle of the graph; the occupations sorted into “used to do” as 2; and the current level of participation or “do now” as “3,” or the outermost ring, the variations in the rings can be seen as differences in occupational participation. More simply, occupations that are currently being done by both groups form the outermost points, while any occupations not touching the outermost circle are those that have been given up or were never done. Further, many of the occupations in which IEH participate are depicted as “used to do,” indicating that these occupations were given up as a result of experiencing homelessness. This validates the importance of including items from the original ACS that may not be as readily applicable to persons currently experiencing homelessness. Qualitative data from the follow-up questions were coded using NVivo and analyzed for themes using conventional content analysis [30, 31].

#### 2.2.1. Results Phase 2

Thirty participants completed the ACS-AIP interview (see Table 2). Several domains of occupation (social, home management, nonsanctioned, and work and education) indicated differences in frequency patterns of occupational participation between groups (see Figure 2). Both groups reported similar participation in the domains of activities of daily living, physical activity, leisure, health management, and community independence. In fact, for the occupations labeled as “nonsanctioned,” both groups reported previous experience participating in these occupations, with both groups reporting drinking alcohol and IEH reporting smoking as current occupations.

Of the domains of occupation wherein IEH reported decreased participation, the majority of participants rated those areas as “desired occupations.” Regarding social occupations, IEH rated the desire to be in a relationship, care for sick loved ones, attend support groups, and care for family as occupations in which they previously participated and desire to participate in again. Under home management, IEH rated food preparation, locating housing, cleaning tasks, and managing mail as previous yet desired occupations. The two groups differed in their participation in work and education occupations. IEH desired to participate in vocational occupations for pay, while nonhomeless participants reported greater participation in educational occupations.

Reflection on the differences between groups provided deeper insight into occupational participation of each population. Participants suggested including missing occupations such as meditating, online shopping, and commuting. The research team determined that all of their suggestions fit into the categories that were already established. As an example, the term commuting was suggested by participants but was determined by the research term to fall under the already existing activities of “driving” and “using public transportation”. Although the majority of participants reported no discomfort using the cards, one respondent suggested that the activity was a reminder of “when my mom was sick...that made me sad. I hate to see her like that.” Another participant reported discomfort talking about substance abuse with members of the research team, who the participant did not know very well. General opinions of the tool were positive, as suggested in these themes: “enjoying thinking about occupations in this way and a good tool for getting a holistic look at occupation.” Overall, homeless and nonhomeless participants reported enjoyment of the tool and feeling that it could be beneficial in identifying future occupation-based goals.

#### 2.2.2. Discussion Phase 2

The aim of this initial construct validity study was to explore whether the occupations present in the ACS-AIP represent the daily experience of IEH and those not experiencing homelessness. In this study, unique occupations reported among homeless participants did not include many of the instrumental activities of daily living and social occupations that nonhomeless participants reported engaging in on a daily basis, despite the desire to participate. However, engagement in many occupations was the same across both groups. This may suggest that the means with which the occupation is performed may be more important than the occupation itself. Thus, individuals across various populations may engage in similar occupations but participate in them in different ways. Generating rapport between the researchers and participants in this study was vital, as the conversation surrounding participation in nonsanctioned occupations is central to planning interventions. Participants who built rapport with the researchers prior to the assessment reported being more comfortable answering honestly about participation in nonsanctioned occupations. Therefore, the therapist–client relationship is an essential consideration when administering this assessment due to the sensitive and self-report nature of many of the items addressed. Additionally, a number of nonsanctioned occupations (e.g., getting drugs and alcohol, stealing, gambling) included in this version of the ACS poses the possibility of retraumatization; therefore, best practices in trauma-informed care should be considered when providing this assessment.

For an individual to achieve a state of health, the opportunity to participate in activities that promote self-confidence, personal identity, and motivation are essential [5, 32, 33]. According to Aldrich [1], “Failing to name an occupation may...signify a lack of awareness of an occupation, a perceived lack of importance attributed to an occupation, or a decision to not address social or political issues related to an occupation” (p. 1). The use of an assessment tool that includes nonsanctioned occupations can bring focus to occupations that are often not acknowledged by occupational therapy practitioners within the profession and provide the clinician with a tool to engage in conversations that previously may have been difficult to initiate yet central to the assessment and intervention process.

#### 2.2.3. Limitations Phase 2

This study focused on a specific population with a limited sample size. Therefore, results are not generalizable to other populations. However, this study provided preliminary data to proceed with further studies and interest of others in developing the ACS-AIP into a valid and useable tool.

Regarding participants, all IEH participants were already seeking services at the shelter, which could have led to a misrepresentation of the occupations in which they engage as opposed to IEH who do not already have access to services at a shelter. A much more diverse and larger sample of nonhomeless participants must also be included in future studies.

## 3. Implications for Occupational Therapy

Occupational therapy practitioners use their knowledge of the relationship between the person, their engagement in meaningful occupation, and their environment to enhance or enable successful participation [5]; however, gaps exist. The ACS-AIP is a unique tool that acknowledges occupational participation patterns in a format that allows for nonbiased exploration of occupations. Thus, implications for occupational therapy regarding the ACS-AIP include:

- Identification and open discussion of nonsanctioned occupations during evaluation, treatment, and discharge planning are necessary to address the complex needs of IEH
- In exploring the occupational participation of IEH, it is important to explore not only the occupations occurring, but how an individual is participating in the occupation. It may be that IEH participate in many similar occupations to other populations, yet limited resources require variations in the way occupations are performed. Further studies are needed to explore this idea
- The ACS-AIP allows practitioners to have an open discussion about occupations that may be uncomfortable to address with clients who otherwise would not share
- The ACS-AIP demonstrates the potential for holistically exploring and assessing occupational participation as a means to facilitate dialogue that improves therapeutic relationships and person-centered occupational therapy goals.

## 4. Conclusion

Engagement in nonsanctioned occupations is an important part of individual identity. All individuals participate in occupations that do not directly improve their well-being but hold meaning for them. Thus, it is essential that occupational therapy practitioners begin to identify those occupations and their impact on quality of life. Reducing the stigma associated with nonsanctioned occupations may be the first step to ensuring that they are addressed during the occupational therapy process. This paper presents an assessment tool aimed at identifying the entire scope of occupational participation for IEH. A discussion of intervention strategies for nonsanctioned occupations is beyond the scope of this paper. However, identification of nonsanctioned occupations using the ACS-AIP has the potential to facilitate person-centered interventions, improve occupational participation among marginalized populations, and expand occupational therapy's value by providing a holistic view of occupation in populations where certain aspects of participation are largely overlooked.

## Figures and Tables

**Table 1:** Occupations identified from interview and literature.

	Source	Occupations initially selected	Occupations used
Tools	Activity Card Sort [11]	90	43
	Shelter Assessment [17]	46	31
	Vineland Adaptive Behavior Scales [18]	23	23
	[8]	7	7
	[19]	25	25
	[20]	30	30
	[7]	3	3
	[21]	28	28
	[22]	9	9

Interviews with IEH	Participant semistructured interview	33	33
	Total retrieved	347	
	Number of occupations after removing redundancies	197	

**Table 2:** Demographics of phase 1.

Individuals experiencing homelessness (*N* = 13)			
Age, *n* (%)	Gender, *n* (%)	Race, *n* (%)	Education, *n* (%)
20–40, 4 (30)	Male, 12 (92)	Black, 8 (62)	College, 2 (15)
41–60, 7 (54)	Female, 1 (8)	White, 3 (23)	Some college, 1 (8)
61–70, 2 (15)		NA, 1 (8)	HS/GED, 5 (38)
		Other, 1 (8)	Other 5, (38)

Note. GED: test of general educational development certifying high school academic skills; HS: high school; IEH: individuals experiencing homelessness; NA: Native American.

**Table 3:** Demographics of phase 2.

Individuals experiencing homelessness (*n* = 10)				
Age, *n* (%)	Gender, *n* (%)	Marital status, *n* (%)	Race, *n* (%)	Education, *n* (%)
20–40, 1 (10)	Male, 6 (60)	Single, 5 (50)	White, 4 (40)	High school, 1 (10)
41–60, 7 (70)	Female, 4 (40)	Separated, 4 (40)	Black, 6 (60)	
61–70, 2 (20)		Divorced, 1 (10)		
Nonhomeless participants (*n* = 20)				
20–40, 4 (20)	Male, 10 (50)	Dating, 1 (5)	White, 16 (80)	Some college, 6 (30)
41–60, 14 (70)	Female, 10 (50)	Married, 18 (90)	Black, 3 (15)	Bachelor's, 5 (25)
61–70, 2 (10)		Divorced, 1 (5)	Asian, 1 (5)	Master's, 4 (20) PhD/OTD, 5 (25)

Note. OTD: occupational therapy doctorate; PhD: doctor of philosophy.

## Data Availability

Data available upon request.
